# Engineering Organoid Platforms for Pathogenesis Research

**DOI:** 10.34133/research.1346

**Published:** 2026-08-04

**Authors:** Xiaolu Han, Jun Zhang, Jiahui Bai, Weiming Tian, Jie Zhou, Lihua Ding, Xiang Gao

**Affiliations:** ^1^ National Key Laboratory of Advanced Biotechnology, Academy of Military Medical Sciences, 100071 Beijing, China.; ^2^ School of Life Science and Technology, Harbin Institute of Technology, 150080 Harbin, China.; ^3^ Department of Microbiology, School of Clinical Medicine, Li Ka Shing Faculty of Medicine, The University of Hong Kong, Hong Kong, China.

## Abstract

Emerging and re-emerging infectious diseases ranging from the 1918 H1N1 influenza pandemic to the recent SARS-CoV-2 and monkeypox virus outbreaks continue to pose profound threats to global public health. These crises underscore the critical need for high-fidelity and human-relevant infection models. Organoid technology has emerged as a cornerstone platform for pathogen research by faithfully recapitulating the 3-dimensional architecture and physiological microenvironment of native human tissues in vitro. This review systematically examines the development and structural refinement of organoid-based infection models with an emphasis on evidence-based strategies for stem cell source selection, extracellular matrix optimization, and dynamic culture system engineering. Such advancements enable the robust generation of multi-organ models including respiratory, intestinal, and neural organoids tailored for investigating viral tropism, spatiotemporal infection kinetics, and host immune responses. Furthermore, we evaluate the translational utility of organoids in high-throughput antiviral drug screening and preclinical vaccine assessment. To further enhance physiological relevance and functional fidelity, organoid platforms are being increasingly combined with advanced engineering strategies, including coculture approaches, CRISPR–Cas9-mediated genetic perturbation, engineered microphysiological systems (such as organ-on-a-chip), and 3D bioprinting. These integrated technologies improve biomimicry while expanding experimental controllability and scalability. In addition, we critically examine the major bottlenecks limiting clinical translation and discuss emerging frontiers driven by artificial intelligence and synthetic biology. Through iterative technological refinement and cross-disciplinary convergence, organoids have evolved beyond reductionist in vitro surrogates into physiologically informed and mechanism-driven platforms that advance our understanding of host–pathogen interactions while enhancing global preparedness against emerging pathogens.

## Introduction

**Fig. 1.**
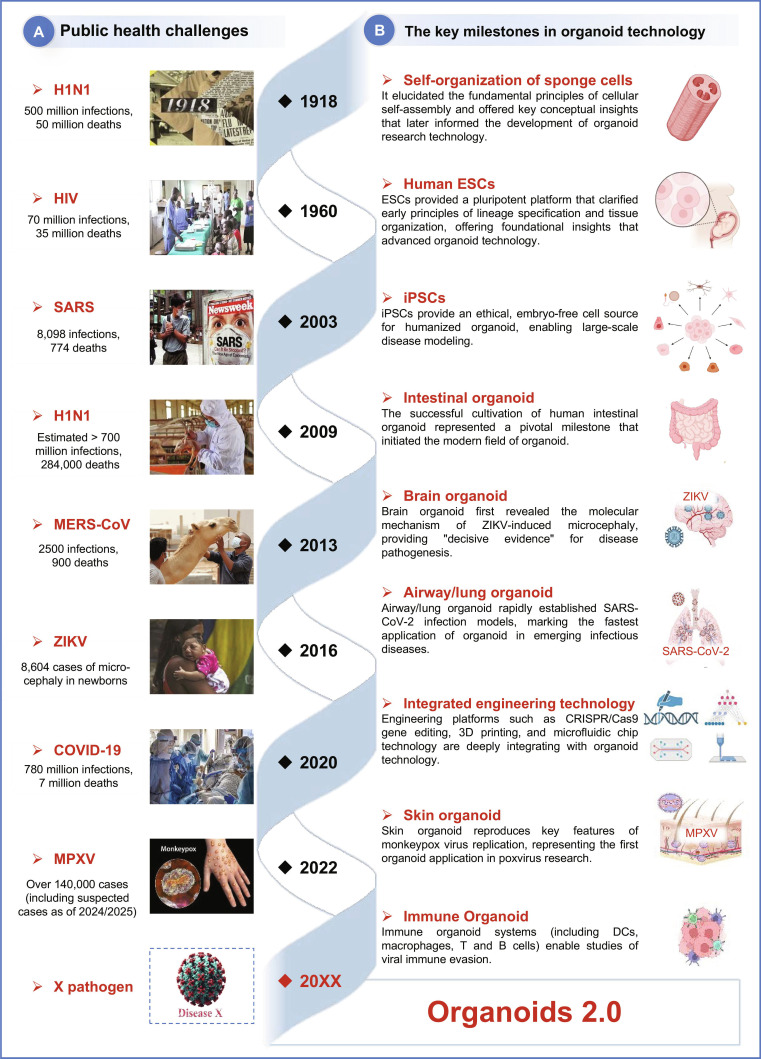
The convergence of public health threats and organoid research breakthroughs. (A) Chronological overview of major global infectious disease outbreaks and their prevalence since 1918. (B) Timeline highlighting pivotal milestones in organoid research and key engineering technologies in pathogen biology. Created with BioRender.com.

The emergence and re-emergence of high-consequence pathogens, including severe acute respiratory syndrome coronavirus 2 (SARS-CoV-2) [1], Ebola virus [2], Marburg virus [3], and highly pathogenic avian influenza [4,5], constitute a persistent threat to global health and public health security (Fig. 1A). The potential infectivity and pathogenicity of these agents and their implications for public health security underscore the urgent necessity for advanced experimental systems capable of precisely simulating human infection dynamics [6]. However, conventional models face inherent limitations in dissecting complex pathogenic processes. Immortalized cell lines lack the essential tissue architecture, cellular heterogeneity, and microenvironmental cues required to mimic cell type-specific defense responses. Similarly, while animal models provide physiological context, they are often confounded by substantial interspecies differences that obscure accurate inferences regarding viral tropism, host range, and immune interactions [7]. These deficiencies not only impede the mechanistic understanding of human-specific infections but also delay the development of targeted medical countermeasures against biological threats.

The advent of organoid technology has catalyzed a paradigm shift in pathogen infection research. Derived from stem cells, these self-organizing 3-dimensional (3D) structures faithfully recapitulate critical features of human tissues, including epithelial polarity, stromal and immune components, and context-dependent signaling networks [8]. By bridging the critical gap between reductionist cell cultures and complex in vivo models, organoids provide a physiologically relevant platform for investigating disease pathogenesis [9]. Since the establishment of human intestinal organoids in 2009, this technology has transformed virology, spanning from the elucidation of Zika virus (ZIKV)-induced microcephaly to the rapid characterization of SARS-CoV-2 tropism and variant fitness during the COVID-19 pandemic [10,11] (Fig. 1B). Notably, recent advancements in barrier and immune-enhanced organoids have further refined the in vitro modeling of infection kinetics, transmission, and immune evasion [12], establishing their indispensable role in revealing pathogenic mechanisms.

Despite these advances, critical knowledge gaps remain regarding tissue-restricted viral dynamics, host determinants of susceptibility, and mechanisms governing cross-species transmission. To address these, the field is increasingly integrating organoid models with CRISPR-based perturbation tools, engineered microphysiological systems (MPSs), and high-dimensional multi-omics profiling. These synergistic approaches facilitate a systems-level characterization of host–pathogen interactions, providing quantitative insights into infection determinants. Consequently, organoids are positioned as a central platform for analyzing pathogen–host dynamics and accelerating the development of therapeutics and preventive strategies against emerging and re-emerging biological threats.

Here, we critically examine the challenges in modeling human-specific pathogen infections and propose strategies to address them through organoid-based systems, with a specific emphasis on their application in pathogen infection research. This review focuses on human-derived models that preserve functional tissue architecture to enable the mechanistic analysis of viral tropism, host susceptibility, and cross-species barriers. By targeting early pathogen–host interactions, encompassing initial entry, cellular reprogramming, and tissue-level responses, the organoid platform provides a robust framework for evaluating medical countermeasures and strengthening global pandemic preparedness capabilities.

## Organoids as Next-Generation Models for Pathogen Research

### Core principles of organoid technology

**Fig. 2.**
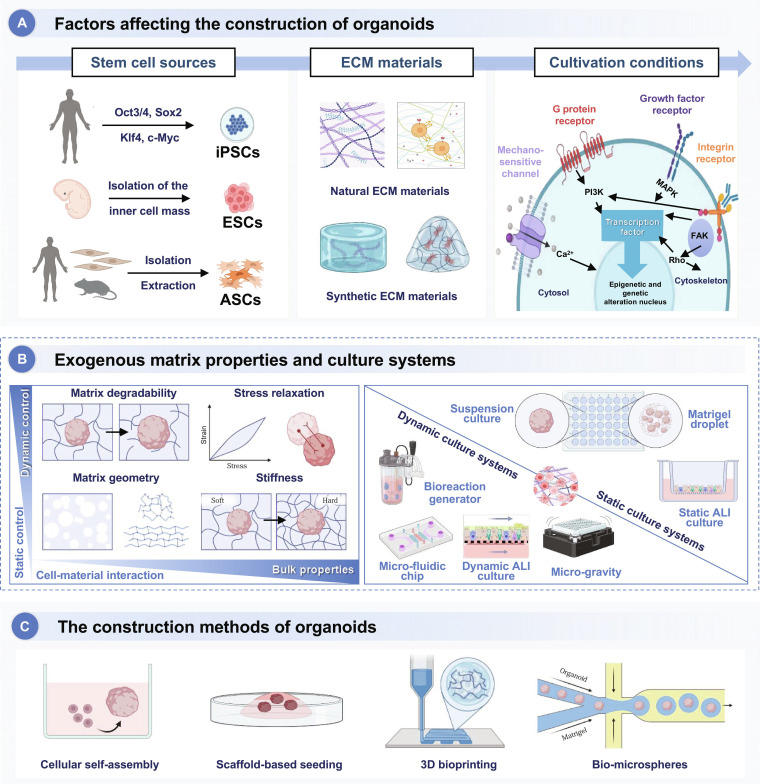
Key factors and methodologies in organoid construction. (A) Core components of organoid establishment, encompassing distinct stem cell sources (e.g., iPSCs, ESCs, and ASCs), ECM scaffolds (natural or synthetic hydrogels), and lineage-specific signaling factors. (B) ECM characteristics and culture system: covering dynamic/static control parameters such as matrix degradation, stress relaxation, and geometric structure, and listing key culture systems such as suspension culture, bioreactor, microfluidic chip, and ALI. (C) The methods for constructing organoids mainly include cell self-assembly, scaffold inoculation, 3D bioprinting, and bio-microsphere technology. Created with BioRender.com.

Organoids are self-organizing, 3D structures generated through the autonomous sorting and lineage-specific differentiation of stem cells under defined culture conditions [13]. These structures faithfully recapitulate the principal cell types of their tissue of origin along with key architectural and functional features (Fig. 2A). This biological fidelity includes the establishment of complex cell–cell interactions and physiological polarity often absent in traditional monolayer cultures. The derivation of organoids relies on distinct stem cell sources. Induced pluripotent stem cells (iPSCs) are reprogrammed via transcription factors such as Oct3/4, Sox2, Klf4, and c-Myc. These cells possess the capacity for unlimited self-renewal and differentiation into all germ layers. Consequently, they are particularly well-suited for generating complex organoids, such as those representing the brain and pancreas, which require intricate developmental cues to form. Pluripotent stem cells (PSCs), encompassing embryonic stem cells (ESCs) and iPSCs, are essential for generating complex multi-lineage organoids. ESCs, derived from blastocysts, exhibit high epigenetic stability and consistent differentiation, making them ideal for establishing high-fidelity baseline models. In contrast, iPSCs enable ethical, donor-specific derivation, which is critical for investigating host genetic determinants of pathogen susceptibility. While iPSCs may retain residual epigenetic memory, their capacity for personalized modeling complements the developmental stability of ESCs, together providing a robust framework for both standardized and population-level pathogenesis research [13]. Conversely, adult stem cells (ASCs) act as tissue-resident progenitors that permit the rapid generation of organoids [14]. Crucially, these ASC-derived models retain the specific genetic and epigenetic signatures of the donor. This unique characteristic provides a robust platform for personalized disease modeling and precision medicine, as it captures the specific susceptibility and immune variations of individual patients.

The extracellular matrix (ECM) provides a critical microenvironment rich in biochemical and physical cues essential for organoid development [15]. Although natural hydrogels, such as Matrigel, facilitate growth, the field is increasingly shifting toward synthetic or defined ECM materials, including polyethylene glycol (PEG) [16], polyacrylamide (PAA) [17], or collagen [18], to overcome batch variability. Natural Matrigel often suffers from undefined compositions and animal-derived contaminants, whereas synthetic alternatives offer tunable mechanical properties and precise chemical definitions. Organoid cells detect physical and chemical cues via G-protein-coupled receptors (GPCRs), growth factor receptors, and integrins [19]. These receptors activate downstream intracellular cascades. Key pathways involve the phosphatidylinositol 3-kinase (PI3K), mitogen-activated protein kinase (MAPK), focal adhesion kinase (FAK), and Rho signaling networks. These cascades regulate the cytoskeleton and drive nuclear transcription factors to determine cell fate and epigenetic states. Ultimately, the precise modulation of these signaling nodes is required to direct stem cells toward specific lineages and ensure the structural and functional maturity of the resulting organoid.

Ideal organoid culture relies on the precise customization of ECM. Physicochemical parameters such as controllable degradability, geometry, stress relaxation properties, and mechanical stiffness collectively determine the adhesion, proliferation, differentiation, and self-organization behavior of stem or progenitor cells, which is central to mimicking the in vivo ECM [20]. In addition to matrix selection, designing the culture system is vital. However, as these advanced engineering approaches become increasingly prevalent, defining the boundaries of these in vitro models ensures terminological clarity. Static methods include submerged organoid culture in hydrogel domes and organoid-derived epithelial models [e.g., air–liquid interface (ALI) cultures] on culture inserts. Notably, these epithelial models, while derived from organoids, lack intrinsic 3D self-organization and are therefore distinguished from bona fide organoids in this review. Hydrogel organoids maintain 3D structure, while ALI models offer well-polarized, differentiated epithelial monolayers with mucociliary or secretory traits, ideal for studying epithelial barrier function and pathogen exposure. Dynamic systems like bioreactors and microfluidic organ-on-a-chip (OoC) platforms provide perfusion flow, mechanical stresses, and precise control over concentration gradients. This improves the simulation of the natural tissue environment and substance exchange. Incorporating organoids or organoid-derived cells into OoC devices creates organoid-integrated MPS, which rely on external engineering rather than self-organization. Integrating matrix science with both static and dynamic culture technologies is essential for creating accurate, functionally mature models, ranging from 3D self-organizing organoids to 2.5D polarized epithelial derivatives and engineered MPS platforms (Fig. 2B).

Current mainstream organoid construction strategies can be broadly classified into 4 major categories, forming a technological spectrum ranging from “bottom-up” self-organization to “top-down” precision manufacturing [21]. Cellular self-assembly is the most classical method, relying on intrinsic interactions between cells and with the matrix to spontaneously form 3D structures, preserving a high degree of self-organization capability but offering relatively lower controllability and consistency. The scaffold-based seeding method involves inoculating cells onto pre-formed biomaterial scaffolds (e.g., hydrogels), providing better spatial guidance and mechanical support, thereby improving structural reproducibility. 3D bioprinting technology represents a more advanced level of engineering control; by sequentially depositing cell-laden bio-inks, it enables high-precision pre-definition of the organoid’s macroscopic structure, cellular composition, and spatial arrangement, paving the way for constructing complex multicellular architectures or vascularized tissue models. Furthermore, bio-microsphere technology generates uniformly sized spherical cell aggregates, providing standardized units for high-throughput drug screening and homogeneous organoid initiation. These methods are not mutually exclusive but are often complementarily integrated based on research objectives, collectively advancing organoid models toward higher complexity, controllable preparation, and standardization (Fig. 2C).

### Organoid models tailored for high-risk pathogen infections

**Fig. 3.**
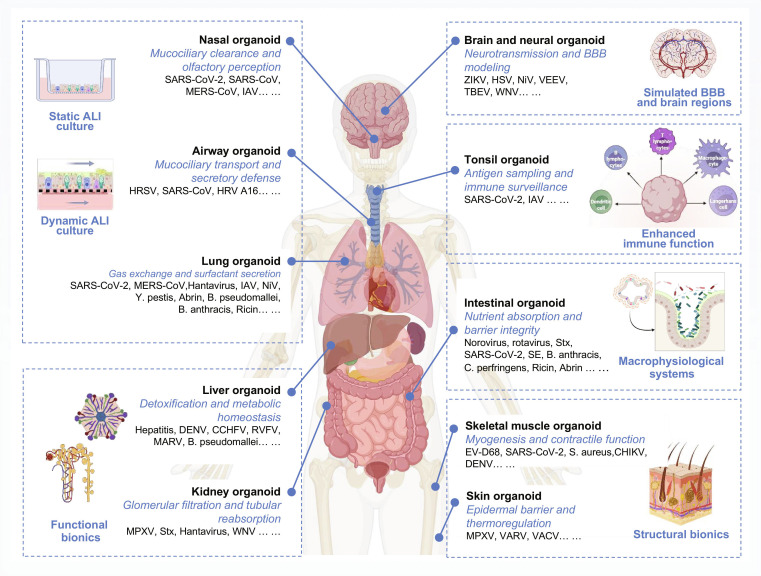
The host–pathogen interface atlas based on organoid models. A systemic map illustrating how organoids from various human organs (e.g., nasal, airway, lung, intestine, liver, kidney, brain, tonsil, and skin) replicate key physiological functions and serve as models for studying infection by specific pathogens (including viruses and bacteria). Created with BioRender.com.

Organoids have emerged as pivotal platforms for elucidating pathogenic mechanisms and assessing biological threats. The organoid-derived infection phenotypes have demonstrated strong concordance with human clinical observations that traditional models often fail to capture. For example, intestinal organoids infected with SARS-CoV-2 accurately replicate the enterocyte damage and interferon responses observed in COVID-19 patients with gastrointestinal symptoms [22]. Similarly, brain organoids exposed to ZIKV effectively mimic the depletion of neural progenitors and cortical disruption associated with neonatal microcephaly [11], neuropathological characteristics that rodent models fail to replicate adequately due to differences in cortical development programs. Organoids offer 3 main advantages over animal models for infection research: (a) They express human-specific receptors like ACE2 and DPP4 with physiological polarity, allowing precise tropism modeling without interspecies receptor differences [7]; (b) they reflect donor-derived genetic diversity, capturing population-level variations in immune response and pathogen susceptibility; and (c) they provide cell type-specific infection data, revealing individual cellular roles in pathogenesis, which are obscured in whole-animal studies. These structural and functional characteristics enable the accurate recapitulation of the host–pathogen interface, thereby substantially enhancing the precision and biological relevance of studies investigating viral and bacterial pathogenesis (Fig. 3).

Respiratory organoids serve as indispensable experimental platforms for the investigation of major respiratory pathogens, encompassing SARS-CoV-2 [10], Middle East respiratory syndrome coronavirus (MERS-CoV) [23], and highly pathogenic influenza viruses [24]. The development of a bipotential organoid has substantially advanced SARS-CoV-2 research by facilitating the concurrent generation of both airway and alveolar lineages from a singular progenitor source. In particular, alveolar organoids, which consist of alveolar type I (AT1) and type II (AT2) cells, have demonstrated efficacy in supporting viral replication [25]. These models have been crucial in elucidating cell type-specific innate immune responses and stress-signaling pathways activated by infection. In parallel, organoid-derived epithelial models such as ALI cultures offer superior physiological fidelity compared to submerged monolayer systems. By mimicking the natural air-exposed environment of the respiratory tract, these systems accurately recapitulate the high infectivity profiles of variants such as Omicron [25] and provide a robust platform for the preclinical evaluation of therapeutic candidates [26].

Digestive tract organoids have effectively resolved the long-standing challenge of culturing noroviruses in vitro, thereby establishing a critical platform for the investigation of viral replication, innate immune defense mechanisms, and the screening of antiviral therapeutics. Furthermore, the integration of CRISPR-Cas9 gene editing with RNA sequencing has empowered researchers to delineate host antiviral signaling pathways with high resolution [27]. In parallel, liver organoids derived from fetal or adult hepatic tissues have successfully recapitulated the complete lifecycle of the hepatitis E virus, revealing that viral progeny is preferentially released via the apical cellular surface [28]. Collectively, these advanced models are indispensable for elucidating the intricate dynamics of virus–host interactions.

Neural organoids, serving as sophisticated 3D models that faithfully recapitulate human brain development, have established themselves as a platform for the investigation of neurotropic viruses. Research utilizing these systems has elucidated that ZIKV infection induces genome-wide DNA methylation alterations across neural progenitor cells, astrocytes, and neurons [11]. These epigenetic modifications are critically associated with the delayed onset of neurological sequelae. Furthermore, these models effectively simulate the neurotropism and structural disruption caused by other important pathogens, including SARS-CoV-2 [29] and human cytomegalovirus [30]. While standard neural organoid protocols lack microglia and a functional blood–brain barrier (BBB), recent advances in MPS have begun to address this gap. BBB-on-a-chip and related human in vitro barrier models have been used to study viral neuroinvasion, barrier dysfunction, and neuroinflammatory responses. For example, SARS-CoV-2 has been modeled in a lung–brain MPS [31], whereas ZIKV has been shown to infect and traverse human BBB models and to disrupt barrier integrity [32]. The integration of neural organoids with BBB chip technology presents a complementary approach for analyzing the entire spectrum of central nervous system infection, encompassing the initial penetration of the barrier to the subsequent pathogenesis within the parenchyma [33].

Vascular organoids effectively address the inherent limitations of traditional models in simulating human vascular dynamics, particularly in response to high-consequence pathogens such as the Crimean–Congo hemorrhagic fever virus (CCHFV), a biosafety level 4 agent. Emerging MPSs, specifically OoC platforms that incorporate vascular and immune components, provide robust environments for simulating intricate virus–vessel interactions [34]. These advanced models faithfully reproduce key pathological processes, including endothelial cell damage, coagulation abnormalities, and immune dysregulation. Consequently, they substantially deepen our mechanistic understanding of pathogenesis and serve as efficient, high-fidelity preclinical systems for the development of novel diagnostics, antivirals, and vaccines.

Musculoskeletal organoids represent an emerging frontier in infection modeling. Bone and joint infections, particularly *Staphylococcus aureus*-mediated osteomyelitis, carry high risks of chronic recurrence and disability, yet conventional animal models poorly recapitulate human bone remodeling and immune dynamics. Recent advancements have facilitated the development of bone organoids derived from mesenchymal stem cells or iPSCs, which self-organize into mineralized structures comprising osteoblasts, osteocytes, and stromal components [35]. These organoids serve as platforms for examining bacterial invasion, biofilm formation, and infection-induced bone degradation. Concurrently, 3D skeletal muscle models derived from iPSCs have elucidated the viral tropism of enterovirus D68 (EV-D68) for human myofibers. EV-D68 infects tissue-engineered skeletal muscles through sialic acid-mediated entry, resulting in tissue damage, reduced contractile force, and impaired muscle regeneration. These findings shed light on the muscular pathogenesis associated with EV-D68-induced acute flaccid myelitis [36]. Similarly, SARS-CoV-2 has been shown to induce myofiber atrophy with persistent metabolic suppression through tumor necrosis factor-α (TNF-α)/nuclear factor κB (NF-κB) signaling, consistent with the long COVID myalgia phenotype [37]. Although still at an early stage, musculoskeletal organoids promise to fill a critical gap in modeling infection-associated tissue destruction and evaluating antimicrobial strategies.

### Quality control and standardization for organoid research

Reliable pathogen research demands rigorous quality control to ensure organoid reproducibility. Standardizing culture media is critical because variations in growth factors and serum alter differentiation. This necessitates chemically defined and xeno-free formulations. Furthermore, standardizing culture conditions is vital for consistent functional maturity. This process requires transitioning from undefined matrices such as Matrigel to synthetic alternatives. Rigorous pre-infection assessment of organoid fidelity is equally essential. Key benchmarks must include morphological criteria, cellular validation through immunostaining or transcriptomics, functional assays such as transepithelial electrical resistance (TEER; a measure of epithelial barrier integrity), and genomic stability monitoring. Establishing these standardized frameworks ensures that organoid-derived data are reproducible, comparable, and acceptable for regulatory evaluation [38].

## Recapitulating Pathogen Infection Dynamics in Organoids

Organoid technology is characterized by its biomimetic 3D structure and presents notable advantages over conventional 2D cultures for investigating pathogen infection and host immune responses. Derived from human stem cells, organoids preserve the receptor diversity, cellular heterogeneity, and spatial organization inherent to native tissues. Consequently, they facilitate more precise modeling of viral entry, replication, and host–pathogen interactions.

**Fig. 4.**
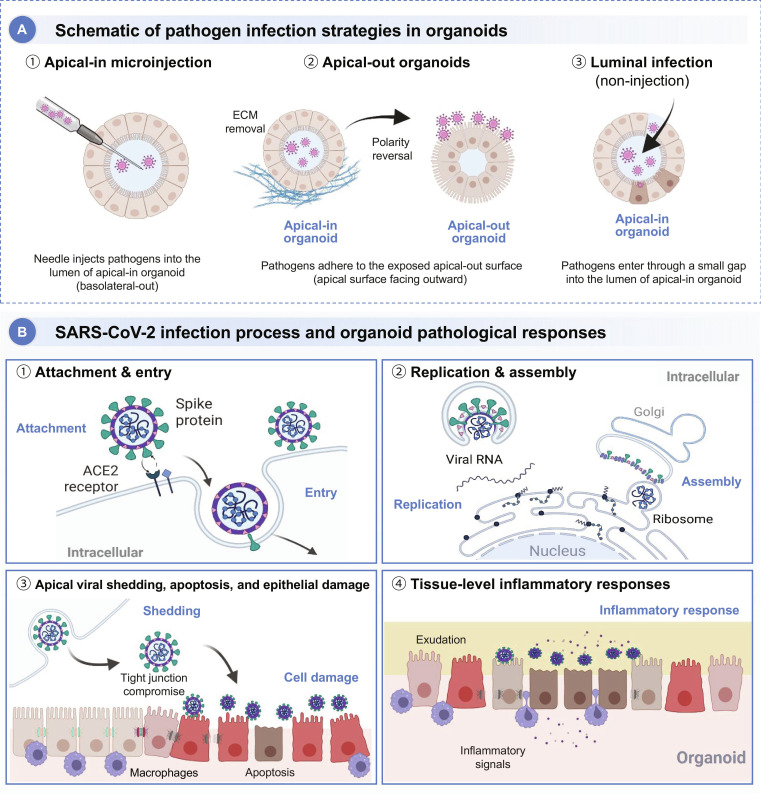
Organoid-based modeling of viral infection dynamics and host responses. (A) Schematic of pathogen infection strategies in organoids, including apical-in microinjection, apical-out polarity reversal, and luminal infection approaches. (B) SARS-CoV-2 infection cascade in polarized organoids: viral attachment to host receptors (e.g., ACE2) and entry via endocytosis; intracellular RNA release, replication, and assembly in endoplasmic reticulum (ER)–Golgi compartments; apical viral shedding with tight junction disruption, apoptosis, and epithelial damage; followed by tissue-level inflammatory responses involving immune cell recruitment, cytokine secretion, and barrier dysfunction. Created with BioRender.com.

Elucidating the directionality of infection is a critical factor in understanding pathogen entry and dissemination. Apical infection mimics pathogen entry through luminal surfaces, including the respiratory or intestinal tracts [23]. Conversely, basal infection involves the breach of epithelial barriers or dissemination via the bloodstream [39]. Luminal infection replicates the microenvironment of hollow organs, exemplified by the gastrointestinal tract. The polarized architecture of organoids features distinct apical and basal surfaces that accurately replicate physiological barriers like mucosal surfaces [40]. However, traditional “apical-in” organoids position their functional surfaces internally and restrict direct access to pathogens. In such systems, microinjection has become a commonly used strategy to introduce pathogens or their products into the organoid lumen, enabling controlled apical exposure and better recapitulating the route of infection in hollow organs. Furthermore, the “polarity reversal” technique addresses this limitation by creating “apical-out” organoids that expose the apical surface to the external environment. This structural inversion markedly enhances experimental accessibility [41]. Mechanistically, this technique utilizes signaling molecules such as lysophosphatidic acid and sphingosine-1-phosphate to activate GPCRs. This activation initiates the RhoA–ROCK signaling pathway and induces cytoskeletal rearrangements to facilitate controlled and reversible polarity switching [42]. This mechanism has been successfully validated in organoids derived from intestinal, cerebral, and pulmonary tissues (Fig. 4A).

To guide researchers in selecting the most appropriate infection strategy, the key technical and biological attributes of the 3 major approaches including microinjection, apical exposure with polarity reversal, and non-injection apical-in infection are summarized in Table 1. The table compares each method across 4 dimensions including organoid polarity, physiological relevance, operational difficulty, and applicable pathogens, thereby providing a practical reference for model selection based on pathogen-specific invasion strategies.

**Table 1.** Major pathogen infection strategies in organoid models: microinjection, polarity reversal, and non-injection luminal infection

Infection strategy	Organoid polarity	Physiological relevance	Operational difficulty	Applicable pathogens
Microinjection	Apical-in	➢ High physiological fidelity ➢ Directly delivers pathogens into the enclosed lumen, enabling controlled exposure to the native apical surface while preserving epithelial polarity and tissue architecture	➢ Technically demanding ➢ Requires specialized micromanipulation equipment and operator expertise, resulting in relatively low throughput	Pathogens requiring precise intraluminal delivery, including enteric viruses, enteric bacteria, and stage-specific parasites
Apical exposure (polarity reversal)	Apical-out	➢ Enhanced biomimicry for apical infection ➢ Exposes the apical surface directly to the culture medium, allowing pathogens to access the physiological entry site without physical barriers	➢ Moderate ➢ Requires ECM removal and specialized suspension culture conditions to establish stable polarity reversal	Pathogens that initiate infection through the apical surface, including respiratory viruses, enteric viruses, bacteria, and other mucosal pathogens
Infection (non-injection)	Apical-in	➢ Mimics natural luminal exposure while maintaining native organoid architecture and polarity ➢ Allows pathogens to enter the lumen through transient openings or epithelial discontinuities and interact with the apical surface	➢ Minimal to moderate ➢ Does not require micromanipulation equipment, but efficient luminal access may depend on organoid morphology and culture conditions	Enteric and mucosal pathogens that naturally infect from the luminal side but do not require precise microinjection

### Pathological foundations of infection models

Pathogen characteristics determine the design logic of organoid models. Viruses are obligate intracellular parasites that rely on specific receptors such as SARS-CoV-2 binding to ACE2 for entry. This requirement necessitates organoid systems that accurately recapitulate cellular tropism and receptor expression patterns, enabling faithful modeling of viral entry, replication, and host responses, as shown in Fig. 4B. In contrast, bacteria often cause disease through extracellular proliferation or toxin secretion, processes that necessitate models with mature mucosal barriers and accessible luminal spaces. Fungal and parasitic pathogens, however, introduce distinct structural and functional requirements. Fungal pathogenesis typically involves epithelial adhesion, hyphal invasion, the secretion of virulence factors, and barrier translocation. Consequently, relevant organoid models must preserve mucosal polarity, epithelial barrier integrity, and, where practicable, host immune components. Parasites, on the other hand, frequently exhibit stage-specific tropism and prolonged intracellular development. For example, the malaria parasite necessitates hepatic platforms characterized by polarized hepatocyte functions and long-term viability to support sporozoite invasion and liver-stage development. Thus, fungal and parasitic infection models further highlight the need to customize organoid architecture according to pathogen-specific invasion strategies.

Furthermore, tissue-tropic infections and opportunistic infections have distinct microenvironmental requirements. Specific infections feature clear tissue tropism and require high-fidelity lineage models. Nonspecific infections often exploit compromised host barriers or inflammatory environments. These cases require the integration of immune cocultures or mechanical strain. In summary, no universal model exists. Model architectures, encompassing self-organizing organoids, organoid-derived epithelial systems, and engineered MPS, necessitate customization in accordance with the pathogen’s invasion strategy. For example, respiratory viruses may use apical-out configurations while hematogenous spread requires vascularized models. This framework supports the specific applications discussed in the following sections.

### Viral infections

Organoid-based systems, including self-organizing organoids and organoid-derived epithelial models, have established themselves as robust in vitro platforms for modeling viral entry, replication kinetics, and host cellular responses. Specifically, direct viral exposure is achieved by mechanically shearing 3D organoids or utilizing suspension-cultured apical-out models. Infection dynamics are subsequently quantified using a defined multiplicity of infection (MOI), serial quantitative reverse transcription polymerase chain reaction (qRT-PCR) for RNA kinetics, and plaque assays for infectious progeny. Human gut [22] and airway [43] organoids have proven indispensable in delineating the intestinal tropism of SARS-CoV-2 and the specific mechanisms governing viral replication within lung epithelial cells. Crucially, the specific model configuration and infection route must be carefully matched to the in vivo pathogenic process. For instance, polarity-controlled airway organoids expose receptors on the exterior surface, directly mimicking luminal respiratory infection and viral shedding. Conversely, basolateral exposure of alveolar organoids simulates hematogenous dissemination, revealing alternative entry receptors like ASGR1. These polarity-specific configurations allow researchers to distinguish between primary mucosal entry and systemic viral spread [44]. For standard basal-out organoids with an enclosed apical lumen, precision microinjection delivers pathogens directly into the core. This technique bypasses the basolateral barrier to faithfully simulate natural mucosal infections. Furthermore, it ensures precise control of the infectious dose while preserving the intact 3D architecture and barrier integrity. Beyond coronaviruses, these platforms have been successfully adapted to interrogate the infection dynamics of other high-consequence pathogens, including influenza, Ebola, and Marburg viruses [10,45–47]. Furthermore, bioengineered skeletal muscle organoids have been utilized to investigate the myotropism and localized inflammatory cascades induced by SARS-CoV-2 [37], addressing the critical need to model virus-mediated musculoskeletal morbidities.

Crucially, the establishment of bat-derived respiratory and intestinal organoids has unveiled the distinct mechanisms underlying the broad-spectrum antiviral capacity of reservoir species. These epithelial cells display a unique “pre-activated” innate immune state, characterized by constitutive high expression of type I/III interferons and interferon-stimulated genes (ISGs). This immunological landscape provides a mechanistic explanation for the ability of bats to serve as natural reservoirs for numerous lethal viruses without succumbing to disease. Research indicates that this sustained, yet controlled, state of immune activation effectively restricts early intracellular viral replication. Simultaneously, it mitigates the excessive inflammatory cascades that typically drive tissue damage in nonreservoir hosts, thereby maintaining a delicate balance between viral coexistence and host health [47]. Consequently, these findings establish a novel comparative framework for analyzing virus–host interactions and elucidating the molecular basis of natural antiviral resistance.

### Bacterial and toxin interactions

Organoid models provide biomimetic platforms for investigating bacterial pathogenesis and toxin-mediated damage under physiologically relevant conditions. Bacterial pathogenesis models require defined inoculation densities and are typically evaluated using TEER for barrier integrity and time-lapse morphometry for toxin-induced organoid swelling. In studies of *Vibrio cholerae* and cholera toxin, human intestinal enteroid/organoid models have recapitulated key secretory responses underlying cholera-like diarrhea and have enabled quantitative swelling-based assays for inhibitor testing [48]. Mechanistically, the cholera toxin binds to GM1 receptors present on epithelial cells and leads to elevated intracellular levels of adenosine 3′,5′-monophosphate (cAMP) and guanosine 3′,5′-monophosphate (cGMP). This signaling cascade activates cystic fibrosis transmembrane conductance regulator (CFTR) channels to drive the secretion of chloride ions and water. This process manifests as measurable organoid swelling, a quantitative phenotype that has been effectively utilized for screening multivalent inhibitors targeting cholera toxin activity. Similarly, *Bacillus amyloliquefaciens* SC06 was shown to promote intestinal stem cell proliferation and differentiation via Wnt/β-catenin signaling in a *C. perfringens*-challenged mouse model, with organoid-based assays corroborating epithelial regeneration and barrier repair under toxin-associated injury conditions [49]. Consequently, this activation enhances epithelial regeneration and repair, thereby providing a promising strategy for mitigating intestinal barrier injury. Furthermore, human intestinal organoids have elucidated the cell-specific toxicity of Shiga toxin-producing *Escherichia coli*. These studies revealed that mesenchymal cells exhibit heightened sensitivity to the toxin and that epithelial damage is exacerbated when the toxin is applied to the basolateral surface. Directional exposure is vital for accurately modeling these toxin kinetics; applying Shiga toxin to the basolateral rather than apical surface of intestinal models reveals heightened mesenchymal sensitivity, faithfully recapitulating the tissue-specific vascular damage observed during severe in vivo infection [50]. Collectively, these findings identify the toxin receptor Gb3 as a clear target for inhibitor design [51].

### Parasites and fungi

Substantial advances have been achieved in modeling malaria parasite infections via organoid systems. Regarding the liver stage of the parasite life cycle, conventional 2D cultures and primary hepatocyte models are characterized by functional dedifferentiation and low infection efficiency. Conversely, liver organoids sustain hepatocyte-specific functions over extended periods and support the complete developmental cycle of both *Plasmodium falciparum* and *Plasmodium vivax* from the earliest stages of infection. Crucially, hepatocytes within these 3D models maintain the necessary polarized receptor distribution for sporozoite invasion, facilitating the accurate physiological simulation of the transition from blood-borne infection to intracellular development [52]. Specifically, organoids are inoculated with isolated sporozoites, and intracellular development is longitudinally tracked via high-content immunofluorescence imaging to quantify parasite burden. Furthermore, 3D culture platforms have demonstrated the capacity to recapitulate parasite–liver interactions and effectively evaluate antimalarial compounds [53]. In the context of cerebral malaria, cortical organoid models have demonstrated that heme exposure induces structural disruption and apoptosis, thereby offering a novel in vitro tool for investigating pathogenesis. Regarding fungal infections, advanced human intestinal epithelial models have been used to study *Candida albicans* barrier invasion and translocation. These studies show that candidalysin-mediated damage contributes to epithelial injury, whereas host NF-κB signaling helps maintain barrier integrity and restrict fungal translocation [54].

Crucially, findings derived from these models strongly correlate with human clinical phenotypes. For example, human intestinal organoids accurately predicted the gastrointestinal symptoms of SARS-CoV-2 observed in patients [55]. This highlights their distinct advantage and novelty compared to conventional animal models. While murine models often require transgenic engineering to overcome species-specific receptor mismatches, human organoids naturally express native receptors. This physiological fidelity enables the discovery of human-centric pathogenic mechanisms and immune evasion strategies that are fundamentally absent in standard animal models, thereby effectively bridging the translational gap.

## From Bench to Application: Organoid Platforms in Pathogen Research

**Fig. 5.**
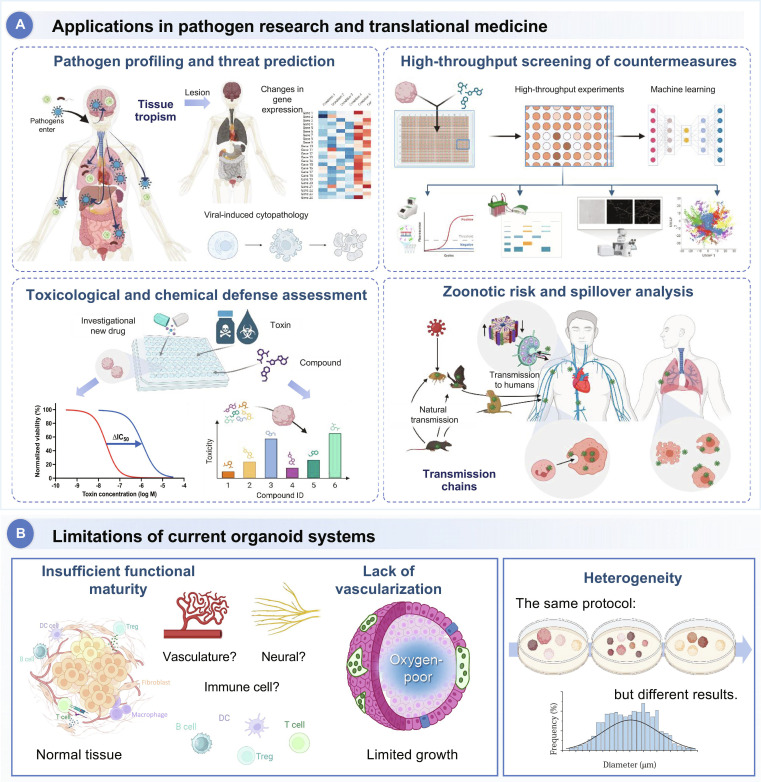
Integrated pathogen assessment pipeline and key limitations. (A) Application directions: pathogen profiling and threat prediction, high-throughput antiviral drug screening, toxicology and chemical defense assessment, and zoonotic disease and spillover risk analysis. These provide essential in vitro models for understanding pathogenesis, accelerating drug development, and enhancing public health preparedness. (B) Major limitations of current organoid platforms include insufficient functional maturity, the absence of resident immune components, diffusion-limited growth, and persistent batch-to-batch variability despite protocol standardization. Created with BioRender.com.

In the domain of public health security, the rapid identification, assessment, and mitigation of biological threats are paramount. By recapitulating key structural and functional features of human tissues, organoid technology offers distinct advantages over traditional models. In particular, it enables systematic investigation of tissue-specific host–pathogen interactions, cellular tropism, and localized pathogenic mechanisms of high-consequence pathogens, and can also support the evaluation of selected aspects of transmission biology and host range that are not readily captured by conventional culture systems. This paradigm shift enables a comprehensive, mechanism-oriented framework for pathogen research, leveraging human organoids to delineate the origins and spatiotemporal dissemination pathways of biological threats and actively bridge the translational gap between benchtop discoveries and clinical interventions (Fig. 5A).

### Pathogen profiling and threat prediction

Emerging infectious diseases necessitate urgent proactive surveillance, as conventional methods often lack the speed to detect the estimated 500,000 unidentified viruses. Organoid-based platforms, including integrated MPS, have the potential to shift infectious disease surveillance from reactive response toward proactive prediction. In contrast to traditional surveillance, organoids simulate authentic infection environments to assess tissue tropism and pathogenesis. For instance, respiratory organoids inoculated with wildlife samples can rapidly identify pathogens with cross-species transmission potential by exhibiting cytopathic effects [56]. This “early warning system” facilitates the preliminary determination of transmission routes. Furthermore, integrating organoids with microfluidic OoC platforms enables the real-time quantification of infection kinetics and cellular damage. This capability yields critical data for estimating disease severity, serving as a foundational element for evidence-based pandemic preparedness strategies.

### High-throughput screening of countermeasures

Contemporary drug development continues to suffer from high attrition rates, due largely to the limited predictive power of conventional preclinical models [57]. Organoid-based systems offer a transformative solution to this “valley of death” by directly bridging the gap between in vitro screening and human clinical trials. By maintaining the inherent heterogeneity of native tissues and preserving microenvironmental signals, organoids markedly enhance the robustness of both efficacy and safety assessments. Although the subsequent examples are not directly related to infectious disease drug development, they demonstrate how evidence derived from organoids and MPS can bolster translational decision-making during the investigational new drug (IND) application stage. HRS-1893, a selective cardiac myosin inhibitor developed for the treatment of hypertrophic cardiomyopathy, particularly its obstructive form [obstructive hypertrophic cardiomyopathy (oHCM)], was evaluated using cardiac organoid-on-chip assays. These assays quantified functional pharmacodynamic parameters, such as contraction amplitude and calcium-transient kinetics, thereby facilitating in vitro activity screening, selectivity assessment, and candidate prioritization before proceeding to in vivo efficacy studies [58]. Similarly, QLF3108, an injectable class I bispecific antibody candidate targeting advanced solid tumors, was assessed using a tumor organoid-on-chip immune coculture platform [59]. This platform reconstituted critical aspects of the human tumor immune microenvironment, allowing for a quantitative evaluation of antitumor activity and elucidation of the mechanism of action, thus providing supportive preclinical evidence for IND-related assessments. Collectively, these examples illustrate the potential of organoid and MPS technologies to enhance the translational research process.

Regarding vaccines, organoids retain native antigen expression and support immune cocultures to simulate adaptive immunity, such as T- and B-cell activation. This capability overcomes traditional limitations in assessing immunogenicity, particularly for mucosal responses to oral vaccines. Recent studies effectively demonstrate this translational utility. For example, immune-competent tonsil organoids successfully recapitulate germinal center formation and secrete specific neutralizing antibodies following influenza vaccination [60]. Similarly, intestinal organoid coculture systems incorporating mucosal immune cells provide a promising platform for modeling local immune responses at the epithelial interface and may support the evaluation of mucosal vaccine-related readouts, including secretory immunoglobulin A (IgA) production and cytokine responses [61]. Furthermore, organoid-based models serve as physiologically relevant platforms for antiviral screening against emerging viral variants and for validating candidate inhibitors in human tissue contexts. Finally, patient-derived models enable the investigation of population-specific infection responses, thereby advancing precision medicine.

### Toxicological and chemical defense assessment

Regarding mechanistic toxicology, organoids recapitulate complex physiological responses often obscured in conventional models. For instance, liver organoids faithfully execute phase I and phase II enzyme-mediated toxin metabolism [62] and pathogen-induced damage [63]. In neurotoxicology, neural organoids and neuromuscular junction models derived from human PSCs have proven instrumental in delineating the molecular mechanisms of neurotoxins, including botulinum neurotoxins [64,65]. Crucially, the integration of these systems with microelectrode arrays permits the precise, quantitative electrophysiological analysis of toxin-induced functional impairments [66]. In the context of precision toxicology, organoids derived from diverse genetic backgrounds enable the investigation of metabolic polymorphisms, thereby supporting individualized risk assessment.

Concerning preclinical safety evaluations, organoids representing primary target organs (heart, liver, kidney) enable the early prediction of cardiotoxicity, hepatotoxicity, and nephrotoxicity. This predictive capability mitigates clinical attrition risks and effectively bridges the interspecies translational gap. Finally, advanced MPS, such as multi-organ-on-a-chip (MOOC) platforms, facilitate multi-tissue linkage, providing comprehensive systemic assessments of agent exposure. Organoid technology promotes the “3Rs principle” (reduction, refinement, and replacement of animals) by offering a scalable platform for analyzing biochemical warfare agent toxicity and developing individualized protection strategies. Furthermore, these platforms support high-content, high-throughput screening, substantially enhancing the standardization and efficiency of testing protocols for chemical warfare agents, thereby providing robust, human-relevant data that regulatory agencies increasingly require for clinical trial authorizations.

### Zoonotic risk and spillover analysis

Zoonotic diseases constitute persistent threats to public health and national defense. By faithfully recapitulating human tissue architecture and receptor profiles, organoid technology provides a critical platform for evaluating the potential of animal-origin viruses to surmount species barriers [56,67]. For instance, comparative analyses using bat and human intestinal organoids facilitate the prediction of coronavirus adaptive evolution and transmission potential.

Specific applications have demonstrated the utility of organoids in risk assessment of cross-species transmission. Studies indicate that human brain organoids exhibit resistance to chronic wasting disease prions, suggesting a robust species barrier [68]. Conversely, pig intestinal organoids show high susceptibility to human coronavirus OC43, providing insights into viral origins and transmission mechanisms [69]. The development of cross-species organoid panels, which include reservoir hosts such as bats and rodents, intermediate hosts like swine and camels, and human tissues, constitutes a robust strategy for systematically mapping spillover risk landscapes. By comparing infection efficiency, receptor utilization, and innate immune activation across species-matched organoids exposed to identical viral isolates, researchers can quantitatively evaluate the adaptive mutations necessary for cross-species transmission and identify molecular determinants that restrict host range. Collectively, these comparative studies are consistent with the “One Health” framework, highlighting how mechanistic insights into pathogen–host interactions may inform broader approaches to zoonotic risk assessment and early warning systems.

### Critical bottlenecks and future translational perspectives

Integrating organoids into clinical pipelines faces major hurdles. Lacking good manufacturing practice (GMP)-compliant manufacturing causes batch variability and hinders regulatory approval. Furthermore, the absent of systemic integration of vascular and immune networks limits accurate pharmacokinetic and pharmacodynamic predictions. Future research must converge organoid technology with microfluidic OoC systems and artificial intelligence (AI)-enabled digital twins. Overcoming these barriers will transform organoids into standardized platforms guiding personalized patient care.

## Hurdles in Translation: Limitations of Organoid-Based Systems

Despite their success in replicating key tissue functions, organoid platforms face some hurdles regarding functional maturity, accessibility, and heterogeneity that constrain their translation to clinical applications (Fig. 5B).

### Insufficient functional maturity and systemic integration

Organoids demonstrate impressive biomimetic capabilities, such as mucus secretion in intestinal models [70] and electrophysiological activity in brain organoids [71]. However, they generally remain functionally immature and lack essential tissue complexity [39,72]. A major limitation is the absence of vascular and neural systems, which hinders the modeling of systemic responses and inter-organ signaling. For example, conventional brain organoids do not possess a functional BBB, which obstructs the study of pathogen entry mechanisms, such as those involving ZIKV, and immune cell infiltration. Although engineering approaches, like the induction of *ETV2* expression, aim to create vascular-like structures, they frequently fail to replicate physiological barrier functions [73]. Similarly, the absence of innervation restricts the exploration of neuro-immune interactions, including the exploitation of neural signaling pathways by pathogens.

Furthermore, the absence of functional vasculature compels organoids to rely on passive diffusion for nutrient and oxygen exchange. This reliance constrains their size to a few hundred micrometers and curtails long-term culture, often compromising the differentiation potential of ASCs. Additionally, PSC-derived organoids typically retain a fetal-like phenotype rather than achieving adult-level maturity. While conventional brain organoids successfully replicate early developmental architecture, they have historically lacked the cellular heterogeneity and electrophysiological complexity required to model mature adult tissues [74]. However, recent breakthroughs have profoundly advanced this paradigm. Most notably, the development of multicellular integrated brain (miBrain) immuno-glial-neurovascular models successfully incorporates all 6 major central nervous system cell types. By co-assembling iPSC-derived neurons, microglia, oligodendrocytes, astrocytes, pericytes, and endothelial cells within an engineered 3D matrix, these advanced platforms overcome previous limitations [75]. This integration successfully captures the multicellular diversity, barrier function, and functional glial-neuronal interactions critical for accurately modeling mature brain physiology and complex neurodegenerative mechanisms.

### The absence of immune components limits host–pathogen research

The lack of adaptive immune components in standard organoids severely restricts their utility in pathogen defense research [76]. Most models focus on epithelial and stromal architectures, omitting T and B lymphocytes required to study pathogen evasion strategies such as major histocompatibility complex (MHC) modulation and immune signaling suppression. Consequently, these systems cannot reproduce critical immunological events, including cytokine dynamics and immune memory formation [77], which fundamentally undermines their ability to evaluate vaccine immunogenicity.

To address this, researchers are developing “immunized organoid” models via 2 primary approaches: the directed differentiation of PSCs into immune tissues (e.g., thymus and bone marrow) [78] or the engineering of composite systems through coculture with peripheral blood mononuclear cells [79]. Despite these advances, substantial challenges persist, including the functional exhaustion of allogeneic cells in long-term culture, the lack of coordinated interactions among multiple immune populations, and the incomplete maturation of iPSC-derived lymphocytes compared to their primary counterparts.

### Heterogeneity and cost

Organoid formation relies on stochastic self-organization processes that result in substantial batch-to-batch heterogeneity in size, morphology, and cellular composition [80]. This variability markedly compromises the consistency of host–pathogen interactions and signaling responses, thereby complicating data interpretation. Furthermore, the absence of standardized assessment metrics erodes experimental reproducibility and hinders the establishment of robust, large-scale production systems essential for reliable biomedical research.

In parallel, organoid culture is considerably more expensive and labor-intensive than conventional methods. The reliance on costly reagents, including specialized matrices like Matrigel and recombinant growth factors, creates financial barriers and supply instability. Additionally, scaling production for high-throughput applications necessitates substantial investment in automation and bioreactor systems. These economic and technical demands currently limit the widespread adoption and accessibility of organoid technology in resource-constrained settings.

## Advanced and Emerging Technologies for Organoid Platforms

Organoid technology is rapidly evolving through the integration of multidisciplinary engineering approaches, ranging from established technologies in infection research, such as coculture, immune-enhanced organoids, vascularization, and MPS, to emerging frontiers like AI-guided design and synthetic biology.

### Coculture strategy

**Fig. 6.**
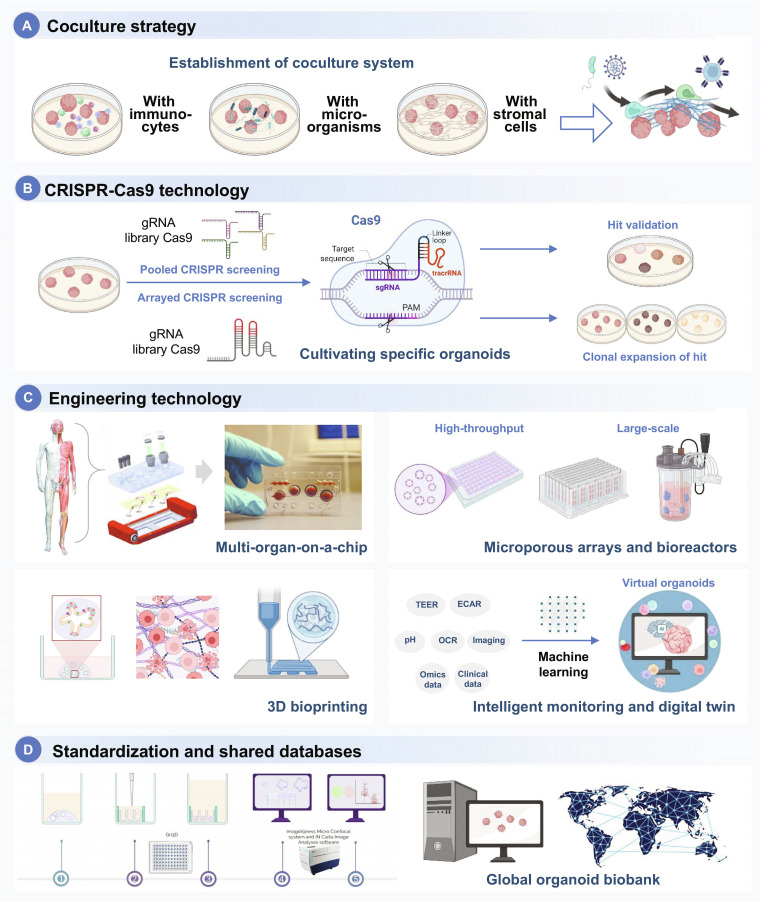
Advanced and emerging technologies for organoid platforms. (A) Coculture strategies integrating immune cells, microorganisms, or stromal cells. (B) Utilization of CRISPR-Cas9 technology for genetic screening and engineering in organoids. (C) Engineering platforms including multi-organ chips, 3D bioprinting, high-throughput microporous arrays, bioreactors, and intelligent monitoring systems. (D) Pathways toward standardization and the establishment of shared global data. Created with BioRender.com.

Coculture strategies applied to organoid-based systems fundamentally reconstruct the complex in vivo microenvironment by integrating multiple distinct cell types into a unified system. This methodological advancement effectively overcomes the inherent structural and functional limitations of single-lineage organoids that lack critical tissue niche components. By recapitulating the multicellular composition of native organs, this approach enables the precise replication of intricate signaling networks and dynamic cell–cell crosstalk mechanisms. Researchers can now simulate physiological interactions by combining organoid units with diverse cellular counterparts. The incorporation of immune cells, including macrophages and lymphocytes, facilitates the modeling of inflammatory responses and immune surveillance. Simultaneously, the inclusion of stromal cells provides essential structural support and regulates epithelial homeostasis through the production of ECM components. Furthermore, the introduction of microorganisms allows for the detailed investigation of commensal–pathogen dynamics and their profound impact on host tissue integrity (Fig. 6A).

Immune-enhanced organoids represent a strategic advancement designed to overcome the critical absence of native immune competency in conventional culture systems. Researchers primarily employ 2 distinct methodologies to integrate immune components. The first approach involves direct coculture. In this configuration, immune cells and organoids are physically integrated within a shared hydrogel matrix. This spatial proximity facilitates the investigation of contact-dependent mechanisms, including the infiltration of immune cells into tissue parenchyma and direct cytotoxic engagement. Alternatively, for organoid-derived epithelial models, indirect Transwell systems utilize a semi-permeable membrane to physically separate cell populations while permitting fluidic communication. This configuration is essential for isolating the role of soluble mediators, such as cytokines and chemokines, in regulating tissue responses without the confounding variables of physical contact. Collectively, these sophisticated platforms enable the real-time visualization of dynamic host defense processes, ranging from pathogen recognition and phagocytic clearance to viral immune evasion strategies. For instance, the development of macrophage-enriched liver organoids, known as MaugO, serves as a prominent proof of concept. These models successfully recapitulate complex pathological events during hepatitis E and SARS-CoV-2 infections. Observed phenomena include robust immune activation, the release of inflammatory cytokines, and subsequent tissue damage. Consequently, such systems demonstrate substantial translational potential for dissecting disease mechanisms and validating multi-target therapeutics [81].

Vascularized organoids address the absence of functional circulatory systems in traditional models through the implementation of diverse bioengineering strategies. One primary method involves the coculture of organ-specific cells with endothelial cells, such as human umbilical vein endothelial cells (HUVECs) or iPSC-derived endothelial cells [82]. This approach promotes the spontaneous self-organization of primitive vascular networks within the organoid structure. Alternative strategies include the simultaneous differentiation of PSCs into both organ-specific lineages and endothelial populations within a single culture system, or the fusion of pre-engineered vascular units with target organoids to rapidly establish perfusion-ready vasculature [83]. Furthermore, advanced engineering methodologies, including 3D bioprinting and microfluidic technologies, facilitate the fabrication of highly biomimetic vascular architectures with tunable geometries and flow characteristics [82,84,85]. The integration of functional vasculature substantially improves the physiological relevance of these models by enabling the precise recapitulation of hematogenous infection routes [86]. By simulating dynamic hemodynamic environments, these systems allow for the real-time monitoring of critical pathological processes. As established state-of-the-art engineering tools, these strategies are now effectively utilized to study systemic pathogen dissemination and the active recruitment of immune cells across endothelial barriers during infection. Recent studies have successfully utilized these platforms to model the recruitment of circulating immune cells, the specific adhesion of pathogens to endothelial barriers, and their subsequent transmigration into the tissue parenchyma [87,88]. Such capabilities are essential for elucidating the mechanisms of tissue-specific pathogen dissemination and understanding the complex roles of tissue-resident immune cells in defense and disease progression.

Organoid–pathogen coculture represents a sophisticated experimental paradigm designed to elucidate the complex mechanisms of viral entry, replication, and subsequent therapeutic responses. This system replicates distinct infection modalities through 3 primary methodological approaches. The first is direct infection, where pathogens are introduced into the culture medium to mimic natural exposure routes. The second utilizes microinjection techniques to deliver pathogens with high precision directly into the luminal compartments of hollow organoids [89]. This method effectively simulates infections that originate from mucosal surfaces. The third approach employs microfluidic aerosolization to generate pathogen-laden droplets. This technique accurately simulates airborne transmission dynamics and respiratory exposure [90]. Collectively, these diverse models have yielded substantial advances in dissecting intricate host–pathogen interactions. Furthermore, they serve as high-fidelity platforms for accelerating the preclinical development and validation of next-generation vaccines and broad-spectrum antiviral therapeutics.

### CRISPR-Cas9 technology

The ongoing progress in CRISPR-Cas gene editing technology has substantially augmented researchers’ capacity to accurately manipulate organoid genomes. CRISPR-Cas9 has become the predominant platform for genome-wide screening, attributed to its simplicity and design flexibility. This system facilitates precise genetic modifications, such as gene knockouts, knock-ins, and point mutations, by employing single-guide RNA (sgRNA) to direct the Cas9 nuclease to specific DNA sequences for cleavage. Consequently, it is now extensively utilized in organoid models [91]. CRISPR-Cas9 now supports knockout/knock-in editing in tissue- and PSC-derived organoids, thereby enabling mechanistic dissection of viral receptors and host dependency factors. In addition to traditional gene knockout/knock-in, the CRISPR-Cas9 technology can also be used to specifically control gene expression. By modifying the Cas9 protein and fusing it with transcriptional repression/activation domains, the expression level of the target gene can be regulated without altering the DNA sequence. This technique is particularly suitable for studying the dynamics of signaling pathways, as it allows for the dynamic adjustment of the expression levels of signaling molecules and the observation of the responses of organoids to such changes (Fig. 6B).

In tissue-derived organoids, a stable Cas9-expressing line is typically established first, followed by lentiviral delivery of individual sgRNAs under optimized conditions to single cells, ultimately generating clonal organoids composed of genetically identical cells. For PSC-derived organoids, inducible Cas9-expressing PSC lines are commonly employed, with gRNAs introduced at the stem cell stage, and edited cells subsequently self-organizing into mosaic organoids during differentiation, with Cas9 activation restricted to defined developmental time windows to enable spatiotemporally controlled genome editing. Despite these advances, challenges remain, including the trade-off between transduction efficiency and organoid viability, off-target effects of CRISPR-Cas9, and intrinsic organoid heterogeneity, which may compromise consistency and reproducibility of editing outcomes [92].

Gene-edited organoids effectively recapitulate interactions between pathogens and host cells, serving as unique, physiologically relevant model systems for elucidating infection mechanisms and advancing antiviral therapeutic strategies. CRISPR-Cas9-mediated knockout of host genes essential for viral entry enables direct validation of receptor functionality during infection, enabling systematic dissection of viral invasion pathways [92]. For example, large-scale gene editing applied to intestinal organoid biobanks integrated with transcriptomic analyses has revealed that SARS-CoV-2 replication is independent of endosomal cathepsin B/L but critically depends on the cell surface protease *TMPRSS2 *[93]. Conversely, by introducing human viral receptor genes into nonsusceptible animal-derived organoids, these models can be rendered susceptible to infection, establishing experimental platforms for investigating cross-species transmission mechanisms and assessing public health risks. Furthermore, genome-wide CRISPR screens can identify unknown host factors in an unbiased manner [94].

### Engineering-enabled organoid technologies

Complementing genome engineering and emerging synthetic biology strategies, organoid technology has further advanced through the integration of micro-nano fabrication, materials science, biomechanics, fluid dynamics, and intelligent sensing [43]. By integrating multiple engineering approaches in a synergistic manner, organoid technology has enabled the creation of a highly controllable physicochemical microenvironment that supports the in vitro development and functional maturation of organoids, thereby substantially improving the reproducibility and scalability of the system. In infection biology, this advanced organoid platform has emerged as a powerful tool for dissecting signal transduction networks underlying pathogen–host interactions and identifying novel therapeutic targets (Fig. 6C).

OoC technology, a class of engineered MPS, leverages microfluidic systems to precisely control nutrient, oxygen, and cytokine gradients, closely mimicking human physiological environments [34,95]. By incorporating mechanical cues such as fluidic shear stress and cyclic strain, these models offer unique insights into infection dynamics that static cultures cannot easily capture. Representative examples such as airway-on-a-chip models used to simulate SARS-CoV-2 infection under mechanical strain, revealing how breathing forces modulate viral entry and subsequent endothelial inflammation. Similarly, gut-on-a-chip platforms integrating parallel epithelial and vascular channels have successfully elucidated the pathogenesis of enteric bacteria by tracking real-time barrier disruption and the active transmigration of immune cells [96]. These systems recapitulate epithelial–vascular crosstalk, offering an ideal tool to study systemic signaling pathways. In pathogen research, OoC’s closed design enhances safety for studying high-risk pathogens (e.g., SARS-CoV-2 and Ebola) by reducing reliance on animal models and minimizing species-specific discrepancies. By interconnecting multiple organ modules via microfluidic channels, OoC enables analysis of pathogen transmission across tissues and the systemic regulation of metabolic pathways. Precise control over flow rates and shear forces further uncovers mechanical signaling roles in tissue homeostasis.

MOOC systems represent a critical step toward “human-on-a-chip” models [31]. These platforms incorporate organoids derived from critical respiratory regions, including nasal, bronchial, and alveolar tissues, into a unified fluidic system. This configuration establishes biomimetic mucosal interfaces that dynamically emulate the migration of pathogens from the upper to the lower respiratory tract. For instance, 3-chamber airway chips replicate compartmental crosstalk, enabling studies of viral infection mechanisms and accelerated drug screening [97]. MOOCs also model tissue-specific pathogen effects: Lung–intestine [98] and lung–brain chips [31] simulate barrier functions and multi-organ viral dissemination through vascularized perfusion, providing insights into organ-specific responses to therapies.

Bioreactors address growing demands for scalable, uniform organoid production. Stirred-tank systems ensure nutrient homogeneity and oxygenation, enhancing differentiation and scalability, although hydrodynamic stress requires optimization. Rotating wall vessel bioreactors create low-shear microgravity-like conditions, promoting 3D self-assembly but necessitating precise bubble control [99]. Electrical stimulation bioreactors drive functional maturation in excitable tissues (e.g., cardiac sarcomere organization in heart organoids) through controlled pulses [100,101]. Microplate bioreactors enable high-throughput screening via perfusion culture and integrated sensors (e.g., pH and oxygen), supporting long-term organoid coculture and drug testing with batch-to-batch consistency [102].

Bioprinting technologies (e.g., extrusion-based and light-based) allow precise spatial patterning of cells and biomaterials to create complex organoid architectures. Projection-based stereolithography achieves micrometer-scale resolution for vascularized networks and organ-chip devices [103,104], while multi-material printing facilitates microenvironment modulation. Volumetric bioprinting combines real-time imaging with computational modeling to minimize mechanical stress, improving cell viability and functional integration. These advances have yielded physiologically relevant models of liver, heart, kidney, and tumor organoids, including 3D-printed perfusable lung systems that simulate viral infections (e.g., influenza) for barrier transmission studies.

Integrated micro-sensors enable real-time, noninvasive monitoring of organoid physiology, tracking metabolic analytes (glucose, lactate) and electrophysiological activity via transepithelial resistance sensors and multi-electrode arrays [105,106]. These systems provide high-resolution data on organoid responses to pathogens or drugs, reducing reliance on endpoint assays. Beyond these established engineering advances, emerging frontiers are now beginning to reshape the field. AI-driven design enables the construction of virtual and predictive organoid models that can simulate development, infection progression, and treatment responses. Multi-organ integration further extends organoid applications from single-tissue models to interconnected systems capable of capturing inter-organ communication and systemic disease processes [107]. In parallel, synthetic biology approaches offer the possibility of engineering programmable organoids equipped with gene circuits, inducible sensing modules, and reporter systems, enabling real-time monitoring and functional control of infection-related processes. Together, these advances may transform organoids from descriptive experimental models into predictive, integrated, and functionally programmable platforms for infection research and therapeutic discovery [108].

### Standardization and shared databases

Despite considerable promise offered by emerging technological strategies, the organoid field continues to face fundamental challenges: substantial variability in culture protocols, operational procedures, and analytical standards across laboratories, with no internationally harmonized regulatory framework yet established. By establishing unified standards and integrating multi-dimensional data, the value of organoid models in signaling pathway research can be maximized (Fig. 6D).

Standardization serves as the cornerstone for ensuring reproducibility and data comparability. For genetically engineered or synthetic biology-enabled organoids, standardization should also encompass circuit stability, inducible response fidelity, reporter calibration, transgene silencing, and biosafety safeguards during long-term culture and application. It focuses on 2 key areas: first, the standardization of culture processes, involving precise formulation of medium components, rigorous control of culture conditions (e.g., temperature and matrix stiffness), and standardized assays for viability and differentiation markers; second, the standardization of quality control, requiring systematic frameworks to define critical attributes like differentiation maturity, genetic stability, and functional properties such as drug response. Global regulatory bodies are increasingly supportive. Furthermore, major multinational pharmaceutical companies, such as Roche and AstraZeneca, have actively integrated these platforms into their preclinical toxicology and efficacy screening pipelines. Consistent with this trend, the U.S. Food and Drug Administration (FDA) has increasingly encouraged the use of new approach methodologies (NAMs), including organoids and OoC systems, in regulatory science and drug development, particularly as part of broader efforts to reduce animal testing and improve the human relevance of nonclinical evidence [109].

Concurrently, establishing shared databases is essential to break down data silos and maximize utility [110]. Open-access databases that aggregate culture parameters, multi-omics data, morphological/functional characteristics, and drug response profiles can enhance collaborative research and enable large-scale comparative analyses. Diverse models have emerged, including omics-centric repositories (e.g., the European Human Cell Atlas Organoid project), commercial living biobanks, and disease-specific databases [111,112].

Global standardization efforts are accelerating. International standardization efforts are ongoing, with multiple International Organization for Standardization (ISO) draft documents addressing terminology, manufacturing, and quality assessment of organoids, such as colorectal cancer organoids. Engineering milestones, like the establishment of fully automated AI-driven organoid systems, further facilitate the transition from lab to clinic. Looking ahead, interdisciplinary collaboration will focus on developing more complex integrated models and unified standards, thereby strengthening the role of organoids as pivotal tools in drug development, infectious disease research, and personalized medicine.

## Conclusions

Organoid technology has emerged as a transformative paradigm in biomedical research and effectively bridges the historical gap between reductionist cell lines and complex animal models. By accurately replicating human tissue architecture, epithelial polarity, and receptor diversity, organoids facilitate infection modeling across major organ systems, such as respiratory, intestinal, neural, hepatic, vascular, and musculoskeletal, by deliberately aligning model configurations with in vivo pathogenic processes to ensure physiologically accurate results. Beyond advancing mechanistic understanding, organoids offer substantial translational potential as high-throughput platforms for antiviral screening, vaccine evaluation, profiling of unknown pathogens, and assessment of spillover risks. Accordingly, these systems may complement existing preclinical pipelines for infectious disease preparedness, particularly in mechanistic studies and selected screening workflows. Nonetheless, several challenges remain, including functional immaturity, the absence of immune and vascular compartments, batch-to-batch variability, and the lack of standardized quality control frameworks, all of which impede translational fidelity. Addressing these challenges requires the incorporation of advanced enabling technologies, such as immune coculture, engineered MPS, and CRISPR-based perturbation, alongside emerging innovations, including AI-guided design, digital twins, multi-organ integration, and globally standardized biobank ecosystems. This ongoing integration will enhance our understanding of host–pathogen interactions and strengthen global preparedness against future biological threats.
